# Visualization of Murine Intranasal Dosing Efficiency Using Luminescent *Francisella tularensis*: Effect of Instillation Volume and Form of Anesthesia

**DOI:** 10.1371/journal.pone.0031359

**Published:** 2012-02-24

**Authors:** Mark A. Miller, Jennifer M. Stabenow, Jyothi Parvathareddy, Andrew J. Wodowski, Thomas P. Fabrizio, Xiaowen R. Bina, Lillian Zalduondo, James E. Bina

**Affiliations:** 1 The University of Tennessee Health Science Center, Memphis, Tennessee, United States of America; 2 St Jude Children's Research Hospital, Memphis, Tennessee, United States of America; Tulane University, United States of America

## Abstract

Intranasal instillation is a widely used procedure for pneumonic delivery of drugs, vaccine candidates, or infectious agents into the respiratory tract of research mice. However, there is a paucity of published literature describing the efficiency of this delivery technique. In this report we have used the murine model of tularemia, with *Francisella tularensis* live vaccine strain (FTLVS) infection, to evaluate the efficiency of pneumonic delivery via intranasal dosing performed either with differing instillation volumes or different types of anesthesia. FTLVS was rendered luminescent via transformation with a reporter plasmid that constitutively expressed the *Photorhabdus luminescens lux* operon from a *Francisella* promoter. We then used an IVIS Spectrum whole animal imaging system to visualize FT dissemination at various time points following intranasal instillation. We found that instillation of FT in a dose volume of 10 µl routinely resulted in infection of the upper airways but failed to initiate infection of the pulmonary compartment. Efficient delivery of FT into the lungs via intranasal instillation required a dose volume of 50 µl or more. These studies also demonstrated that intranasal instillation was significantly more efficient for pneumonic delivery of FTLVS in mice that had been anesthetized with inhaled (isoflurane) vs. parenteral (ketamine/xylazine) anesthesia. The collective results underscore the need for researchers to consider both the dose volume and the anesthesia type when either performing pneumonic delivery via intranasal instillation, or when comparing studies that employed this technique.

## Introduction

Intranasal instillation is currently the most widely used method for delivery of drugs, vaccines, or pathogen challenges targeted for either the upper respiratory tract (URT) or lungs of research mice. Intubation is an alternative method [1], [2], [3], [4], [5], [6], [7] that allows for very efficient delivery of materials into the lungs, but the procedure is technically much more demanding and more time-consuming than intranasal administration. In addition, intubation includes a much higher risk of injury to the animal that could compromise the study. Aerosol administration via a nebulizer-based device [8], [9], [10], [11] also offers very efficient delivery of materials to the lungs with little risk of injury to the animal; however, this method is technically demanding and requires expensive equipment that is not widely available. Moreover, the use of aerosol generators for studies involving dangerous pathogens involves safety issues for research personnel that do not exist when using the intranasal delivery method. In light of these factors, it seems certain that intranasal administration will continue to be the most popular method for pneumonic instillation for the foreseeable future. Surprisingly, there is a paucity of published literature describing the efficiency of intranasal instillation of drugs, vaccines, or infectious agents. Therefore, an analysis of the efficiency of pneumonic delivery via intranasal instillation that would allow for standardization of the two most critical variables associated with this technique, namely dose volume and type of anesthesia, would be of great benefit to researchers working with murine models.

*Francisella tularensis* (FT) is a gram-negative facultative intracellular bacterium that causes a high morbidity/mortality zoonotic disease known as tularemia. FT is one of the most virulent bacterial pathogens in humans, as evidenced by its published LD_50_ of less then 10 CFU [12], [13]. Because of its high infectivity and the relative ease with which if can be disseminated via the aerosol route, FT is considered to have high potential for use as a biological weapon. The *Francisellaceae* family of bacteria has a single genus, *Francisella*, which has been divided into two species: *Francisella philomiragia* (a muskrat pathogen) and *Francisella tularensis*. *F. tularensis* has been further subdivided into four subspecies: *tularensis* (type A), *holarctica* (type B), *novicida*, and *mediasiatica* [14]. Of these, only subsp. *tularensis* and subsp. *holarctica* cause disease in humans [15]. The live vaccine strain (LVS) of FT (FTLVS) is an attenuated *F. holarctica* strain that was developed as a vaccine candidate in the former Soviet Union [16]. While FTLVS is highly attenuated in humans, it remains virulent in mice and causes a tularemic disease syndrome similar to that observed in humans [17].

Technological advances in small animal imaging have made it possible to monitor in real-time the growth and dissemination of fluorescent or bioluminescent bacteria in individual animals over the entire course of infection, offering a powerful alternative to traditional methodologies. Bioluminescence has proven to be particularly useful for this application. We recently created a novel bioluminescence reporter vector (pXB173-lux) that encodes the *P. luminescens* lux operon downstream of the FT P*gro* promoter. The lux operon contains genes that are required for production of both luciferase and luciferin, and transformation of FTLVS with this vector causes the bacteria to constitutively produce light during *in vitro* or *in vivo* growth [18]. In this report, we employed an IVIS Spectrum whole-animal live imaging system, coupled with bacterial load determinations, to evaluate in real-time the efficiency of intranasal instillation for initiation of lower respiratory tract (LRT) infections in mice. The results presented here provide striking visual evidence that both the instillation volume and the type of anesthesia used during instillation have a significant impact on the efficiency of pulmonary delivery of FTLVS.

## Results

**Figure 1:**
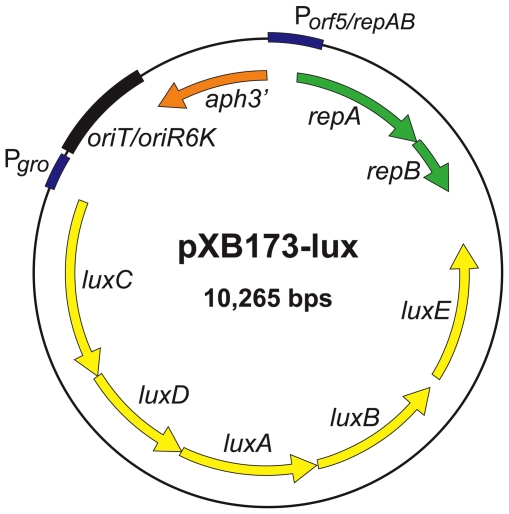
Genetic Map of pXB173-lux. pXB173-lux constitutively expresses the *Photorhabdus luminescens* luciferace (*lux AB*) and luciferase substrate (*luxCDE*) from the *Francisella groE* promoter. This vector also encodes a selectable marker for kanamycin resistance (*aph3′*).

**Figure 2:**
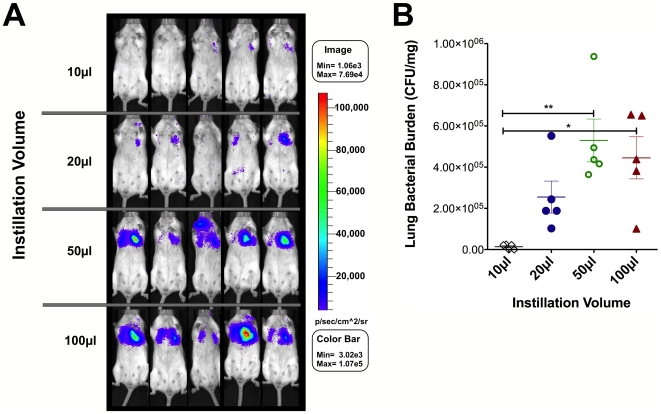
Correlation between whole animal *in vivo* imaging with viable bacterial counts 24 hours after challenge. BALB/c mice (5/group) were challenged with 1×10^6^ CFU of FTLVS bearing the pXB173-lux reporter plasmid via the intranasal route in a total bolus volume of either 10 µl, 20 µl, 50 µl, or 100 µl. **Panel A**: Dissemination of FTLVS was then monitored 24 hrs later using an IVIS Spectrum whole animal imaging system. Images were collected at the indicated time points post-infection and were normalized to reflect photons per second per cm∧2/sr. (**Panel B**): Lungs were collected after imaging was completed for bacterial burden determination via dilution plating. Statistical analyses were performed via one-way ANOVA using a Bonferroni multiple comparisons posttest. Statistically significant differences are indicated as follows: p<0.05 (*) and p<0.01 (**).

To evaluate the efficiency of intranasal administration for delivery of FT to the lungs, BALB/c mice (5/group) were anesthetized with inhaled isoflurane and then challenged with 1×10^6^ CFU of FTLVS bearing a luminescent reporter vector (pXB173-lux, **Figure 1**, see [18]) in a total volume (PBS) of either 10 µl, 20 µl, 50 µl, or 100 µl. Each mouse was subjected to live whole animal luminescent imaging using an IVIS Spectrum imaging system beginning 24 hours post-challenge (**Figure 2A**). The imaging studies revealed a clear difference in luminescent signal from the lungs that correlated with the dose volume used for intranasal instillation. While very little luminescent signal was observed emanating from the lungs of mice challenged using a 10 µl or 20 µl dose, the mice that were dosed using the larger instillation volumes (50 µl or 100 µl) displayed significantly higher levels of luminescent signal. These findings were confirmed by sacrificing the mice immediately after imaging was completed to determine the bacterial burdens in the lungs using standard dilution plating techniques (**Figure 2B**).

**Figure 3:**
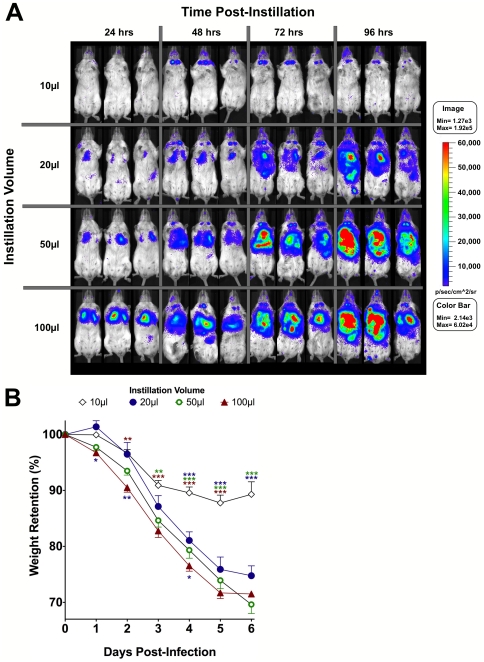
Kinetic *in vivo* localization of luminescent FTLVS following intranasal dosing in titrated volumes of inocula. BALB/c mice (3/group) were challenged via the intranasal route with 1×10^6^ CFU of FTLVS-lux suspended in a volume of 10 µl, 20 µl, 50 µl, or 100 µl of sterile PBS. **Panel A**: All mice were then subjected to whole animal imaging using an IVIS Spectrum Imaging system at the indicated time points. Scaling intensity of all images was normalized and data are reported as photons/sec/cm∧2/sr. **Panel B**: All mice were weighed daily as a measure of disease-state. Statistical analysis was performed via 2-way ANOVA with Bonferroni post-tests. Significant differences between the 10 µl instillation volume group and all other groups are indicated toward the top of the graph and are color-coded. Significant differences between the 100 µl instillation volume group and either the 20 µl or 50 µl dose volume groups are indicated toward the bottom of the graph and are color-coded. The calculated p values are indicated as follows: p<0.05 (*), p<0.01 (**), and p<0.001 (***).

To determine the impact of dose volume-dependent challenge efficiencies on the course of experimental tularemic disease, we performed a kinetic IVIS imaging analysis of BALB/c mice (3/group) were anesthetized with inhaled isoflurane and then challenged with 1×10^6^ FTLVS-lux using a range of instillation volumes (10 µl, 20 µl, 50 µl, or 100 µl). Each mouse was subjected to live whole-animal luminescent imaging at 24 hr intervals post infection. These imaging studies revealed striking differences in both the rate and tissue specificity of FT dissemination (**Figure 3A**). At the 24-hour time point, the luminescence emanating from the lungs of challenged mice confirmed that the larger instillation volumes more efficiently delivered bacteria into the lungs. By 48 hrs post-challenge, luminescent signatures were detected in or around the upper airways of all of the animals. In the mice that were challenged using a 10 µl volume, the luminescent signature remained confined to the upper airways over the entire timecourse, suggesting that the bacteria never disseminated from the upper airways to the lungs, liver or spleen. In contrast, the luminescent signatures observed in the lungs of mice challenged using instillation volumes of 20 µl, 50 µl, and 100 µl increased in intensity over the timecourse. Moreover, the infection appeared to disseminate from the lungs to the liver and spleen in each of these experimental groups, and the rate of dissemination increased with increasing instillation volume used to administer intranasal challenge. Each mouse was also weighed daily as an assessment of disease state. Weight retention results confirmed that the dose volume used for intranasal instillation had a significant impact on the course of tularemic disease (**Figure 3B**). A significant difference (p<0.05) in weight retention between mice that were dosed using a volume of 10 µl vs. 100 µl was observed as early as 2 days post-infection, and by day 4 post-infection there was a highly significant difference (p<0.001) between the 10 µl volume group and each of the other experimental groups.

**Figure 4:**
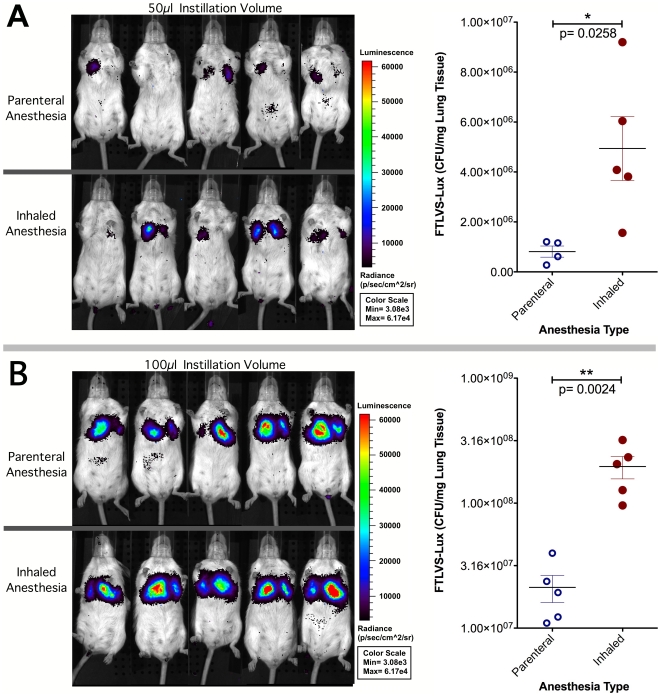
Pulmonary delivery of FTLVS-lux was more efficient under inhaled vs. parenteral anesthesia. BALB/c mice (5/group) were anesthetized using either inhaled isoflurane or parenterally-administered ketamine/xylazine and then challenged with either 1×10^5^ CFU FTLVS-lux in a volume of 50 µl (**Panel A**) or 1×10^6^ CFU FTLVS-lux in a volume of 100 µl (**Panel B**). Dissemination of FTLVS was monitored 24 hrs later using an IVIS Spectrum whole animal imaging system. Lungs were collected after imaging was completed for bacterial burden determination via dilution plating. All IVIS images were normalized to reflect photons per second per cm∧2/sr. Statistical analyses were performed using the student t test.

To examine the efficiency of intranasal dosing for pneumonic delivery under differing forms of anesthesia, BALB/c mice (5/group) that had been anesthetized with either parenteral (ketamine/xylazine) or inhaled (isoflurane) anesthesia were challenged with FTLVS-lux in an instillation volume of either 50 µl or 100 µl. IVIS imaging studies and lung bacterial burden determinations were performed 24 hrs post-infection (**Figure 4**). The intensity of luminescence emanating from the lungs of mice challenged under inhaled anesthesia was more intense than observed in mice anesthetized with parenterally-administered ketamine/xylazine. The bacterial burden in the lungs was then determined via dilution plating. Mice that were anesthetized using inhaled isoflurane had significantly higher bacterial burdens in their lungs than mice that had been anesthetized with parenteral ketamine/xylazine (**Figure 4**), confirming the IVIS imaging results.

## Discussion

Intranasal instillation is the most widely used method for delivery of drugs, vaccines, and/or infectious agents into the respiratory tract of research mice. However, over the years there have only been a handful of published studies designed to analyse the efficiency of intranasal dosing. The primary variables that should be considered when employing this technique are: i) type of diluent used as vehicle for instillation, ii) physical positioning of the mouse during and after instillation, iii) instillation volume, and iv) the type of anesthesia used during instillation. Two of these variables, namely instillation vehicle and physical positioning of the mouse, were not considered in this report. Previously published findings revealed that aqueous diluents were preferable for intranasal instillation [19], therefore, PBS was used as the vehicle for all of the experiments described herein. Moreover, there have been several published studies in which positioning of the mouse during intranasal instillation was considered, and while some of the findings indicated that supine positioning was best for promoting delivery of inocula to the lungs [19], other findings suggested that positioning of the mouse did not have a significant impact on pneumonic delivery efficiency [20]. Therefore, for all of the studies reported here, mice were held in a tilted supine position with their heads elevated to between 60 and 75 degrees above their feet during and after (for approximately 1 minute) instillation.

Dose volume is the best characterized variable associated with intranasal instillation. Published studies have evaluated instillation volumes between 2 µl [21] and 100 µl [22]. Several reports used either dyes or radioactive tracers to investigate the relative efficiencies of various instillation volumes. Visweswaraiah and colleagues performed instillations with Evan's blue dye to evaluate the efficiency of lung delivery using instillation volumes between 5 µl and 50 µl [23] and found that lower instillation volumes resulted in retention of the inoculum in the URT while delivery of dye to the lungs was accomplished only with the larger bolus volumes. A similar study using a radioactive tracer (^99m^Tc-SC) also concluded that instillation in a total volume of 5 µl resulted in retention of the tracer in the URT with no detectable delivery to the lungs [20]. This study also concluded that 35 µl was the optimal instillation volume for delivery of tracer into the lungs. Eyles and colleagues performed instillations of radiolabeled microspheres in either 10 µl or 50 µl volumes and found that the radioactive microspheres accumulated in the URT when delivered in the lower volume while approximately 50% of the microspheres were delivered to the lungs when administered in the larger volume [24]. Because we could find no studies in which efficiency of pneumonic delivery via intranasal instillation was tested using a bacterial agent, we performed a series of intranasal instillation studies using luminescent FTLVS. Our findings were consistent with those employing dyes or radioactive tracers and confirmed that instillation in small volumes (10 µl) resulted in delivery of FTLVS only to the URT while instillation in larger volumes (50–100 µl) resulted in delivery of luminescent bacteria to the lungs.

The use of anesthesia and the type of anesthesia used during intranasal instillation is another variable that could have a significant impact on its efficiency for delivery of inocula to the lungs. It has been previously shown that delivery of materials to the lungs via this technique is significantly more effective when instillation of mice is performed under anesthesia during the procedure [19], [20], [23]. In fact, one of these studies showed that when intranasal instillation is performed on unanesthetized mice, much of the instilled material was delivered to the gastrointestinal tract suggesting that alert mice tend to swallow a significant portion of the inoculum [23]. In light of these findings, and likely because intranasal instillation is technically much easier to perform on anesthetized mice, the majority of researchers using this technique routinely anesthetize mice prior to the procedure. Therefore, it is surprising that there has only been one other published study that examined the effects of different types of anesthesia (parenteral vs. inhaled) on the efficiency of this procedure for delivery of materials to the lower respiratory tract (LRT) [20].

Because isoflurane and ketamine/xylazine are commonly used for anesthesia in rodent-based research [25], we performed a direct comparison of the efficiency of pneumonic delivery of FTLVS-lux via intranasal instillation under these two types of anesthesia. In light of our general observation that mice anesthetized using parenteral-administered ketamine/xylazine maintain a steadier breathing pattern than mice anesthetized for relatively short periods using inhaled isoflurane, we hypothesized that intranasal instillation would be more efficient for pulmonary delivery of bacteria in mice that had been anesthetized with ketamine/xylazine. To our surprise, delivery of bacteria to the lungs was significantly more efficient when instillation was performed under inhaled anesthesia. These results lead us to speculate that the irregular Cheyne-Stokes type respiratory pattern that is typically observed following removal of mice from an induction chamber with inhaled anesthesia causes transient hypoxia which results in deeper inhalation of larger volumes of inoculum per breath, facilitating more efficient delivery of the material to the LRT. In contrast, mice that receive injectable anesthesia breathe in a more-regular and more-shallow pattern, resulting in a more-even coating of the URT surface with the inoculum. Because the inoculum is more distributed along the mucosal surface of the URT and the breathing pattern of the mice is more shallow and regular, the inoculum is not delivered as efficiently to the LRT. Our findings are in contrast to those from the other published study that performed a comparison of inhaled vs. parenteral anesthesia and concluded that the efficiency of pulmonary delivery of intranasally instilled materials was not impacted by the type of anesthesia [20].

It is relatively well established [20], [23], [24], [26], [27] that intranasal instillation for the purpose of URT delivery requires a low administration volume (≤10 µl). However, no consensus has been reached with respect to dose volume for delivery to the LRT. A random survey of very recently published papers in which intranasal instillation was employed for delivery of infectious agents (bacterial and fungal pathogens) to the lungs of mice revealed that researchers used a wide range of dose volumes in combination with a variety of anesthesia types as follows: i) 20 µl instillation volume under pentobarbital [28] or ketamine/xylazine anesthesia [29], [30], [31], [32], [33], ii) 25 µl instillation volume under either pentobarbital [34] or ketamine/xylazine anesthesia [35], iii) 30 µl instillation volume under ketamine/xylazine anesthesia [36], iv) 35 µl instillation volume under ketamine/xylazine anesthesia [37], v) 40 µl instillation volume under isoflurane anesthesia [38], vi) 50 µl instillation volume under either ketamine/xylazine [39], isoflurane [40], [41], [42], [43], [44], halothane [45], [46], and vii) 100 µl instillation volume under isoflurane anesthesia [47]. Moreover, a number of recent papers either supplied information regarding the dose volume used without indicating the type of anesthesia [48], [49], indicated the anesthesia used but failed to indicate the dose volume [50], [51], or supplied no information regarding either the dose volume or anesthesia [52], [53], [54] used for intranasal instillation.

The findings presented here have clearly shown that the efficiency of pneumonic delivery via intranasal instillation is significantly impacted by the volume of the inoculum as well as by the type of anesthesia used during the procedure. These findings underscore the importance of considering both variables when comparing the experimental results between studies that involved intranasal instillation for the purpose of delivering infectious agents or other materials to the lungs. Therefore, it is critical that a complete description of the methods used to anesthetize and inoculate mice via intranasal instillation be supplied in any study that employs this technique. Further studies are needed to determine whether “high-volume” intranasal instillation has any deleterious effects on research mice. The reference book entitled “The Mouse in Biomedical Research” states that intranasal instillation in volumes greater than 20 µl can result in suffocation and death of research mice [55]. While our findings do not support this conclusion, we have noted brief respiratory distress in mice that have received intranasal instillation volumes ≥50 µl. Therefore, studies designed to evaluate respiratory function before, during, and after intranasal instillation with a range of instillation volumes are warranted. We are also initiating studies that will determine whether instillation in larger volumes alters the microenvironment of the lung enough to alter pneumonic disease progression.

## Materials and Methods

### Bacterial Strains and Growth Conditions

*F. tularensis* strain LVS (live vaccine strain) was obtained from the Centers for Disease Control and Prevention (CDC, Atlanta, GA). *F. tularensis* was cultured in modified Mueller Hinton broth (MMH broth supplemented with 10 g/L tryptone, 0.1% glucose, 0.025% ferrous pyrophosphate, 0.1% L-cysteine, and 2.5% calf serum) or on MMH agar (supplemented with 5% calf serum and 1% IsoVitalex). FTLVS was transformed with the luminescence reporter plasmid (pXB173-lux) as described previously [18]. Kanamycin (km) was used at 10 µg/mL to maintain selection for FTLVS bearing the lux-reporter plasmid. We received authorization from the CDC, the Department of Health and Human Services, and from the University of Tennessee Health Science Center (UTHSC) Institutional Biosafety Committee for the use of *aph3′* in FT.

### Mice

Female BALB/c mice were purchased from either Jackson Laboratories or Charles River Laboratories. Mice were age-matched and used between 8 and 16 weeks of age. Mice were housed in an AALAC accredited facility in microisolator cages with food and water available *ad libitum*. All experimental protocols were reviewed and approved by the UTHSC IACUC.

### Anesthesia and Intranasal Instillation

Prior to instillation, mice were anesthetized using either inhaled (isoflurane) or parenteral (ketamine/xylazine) anesthesia. Isoflurane was delivered using a SurgiVet® Vaporstick small animal anesthesia machine equipped with a Classic T3™ isoflurane vaporizer (Smith Medical, Dublin, OH) and mice were exposed to 2.5% isoflurane delivered in O_2_ (2 L/min) within a 1 liter induction chamber until a state of areflexia was reached. Ketamine (KetaVed, VEDCO, St. Joseph, MO; 9 mg/10 g body weight) and xylazine (AnaSed, Lloyd Laboratories, Shenandoah, IA; 1 mg/g body weight) were administered intraperitoneally and mice were challenged once a stable plane of anesthesia was reached. Intranasal administration of each challenge dose was performed by pipetting approximately half of the designated volume of PBS (containing the indicated number of FTLVS) onto the outer edge of each nare of the mice. Mice receiving isoflurane anesthesia were removed from the induction chamber and instillation was performed immediately; no supplemental anesthesia or oxygen was administered following removal from the induction chamber.

### Whole-Animal Luminescent Imaging

The photon emissions from mice that had been infected with FTLVS-lux were measured using an IVIS Spectrum whole live-animal imaging system (Caliper Life Sciences, Hopkinton, MA). Mice were anesthetized with isoflurane using a precision vaporizer and oxygen before and during imaging. Images were collected using medium binning with an F-stop of 1, and the maximum exposure time was 5 minutes. The luminescent signals for all images in any individual study were normalized and reported as photons/second/cm∧2/sr.

### Bacterial Burden Determination

Lungs of challenged mice were removed aseptically and homogenized (using a closed tissue grinder system, Fisher Scientific, Pittsburgh, PA) in one ml of sterile PBS, and the final volume was adjusted to 1.25 ml with PBS. To disrupt cells (releasing FT), 0.25 ml disruption buffer (2.5% saponin, 15% BSA, in PBS) was added with light vortexing. Appropriate dilutions of each sample were then plated in duplicate using an Eddy Jet spiral plater (Neutec Group Inc., Farmingdale, NY) on MMH agar plates and incubated at 37°C for 48–72 hours. Colonies were counted using a Flash & Go automated colony counter (Neutec Group Inc.).

### Statistical Methodology

Statistical analyses of each figure were performed using GraphPad Prism software (GraphPad Software, La Jolla, CA). The specific statistical method used for each dataset is described in the figure legends.
